# Comparative Analysis of Serum Lipid Profiles in Sanctuary-Housed Chimpanzees (*Pan troglodytes verus*) at Tacugama Chimpanzee Sanctuary †

**DOI:** 10.3390/vetsci12100985

**Published:** 2025-10-13

**Authors:** Ethan Renfro, Anneke Moresco, Ismail Hirji, Zoë MacIntyre, Kylie McDaniel, Yedra Feltrer-Rambaud, Thalita Calvi, Larry J. Minter, Aimee Drane, Joshua C. Tremblay, Bala Amarasekaran, Kimberly Ange-van Heugten

**Affiliations:** 1 Department of Animal Science, North Carolina State University, Raleigh, NC 27607, USA; emrenfr2@ncsu.edu (E.R.); jb.minter@nczoo.org (L.J.M.); kdange@ncsu.edu (K.A.-v.H.); 2 Veterinary Technology, Tech, Colorado Mesa University, Grand Junction, CO 81501, USA; 3 International Primate Health and Welfare Group, 28028 Madrid, Spain; yedrafeltrer@hotmail.com (Y.F.-R.); thathacalvi@me.com (T.C.); a.l.drane@swansea.ac.uk (A.D.); jtremb01@alumni.uoguelph.ca (J.C.T.); 4 Tacugama Chimpanzee Sanctuary, Freetown, Sierra Leone; ismailhirji@gmail.com (I.H.); zoemac59@gmail.com (Z.M.); kyliemcdaniel17@gmail.com (K.M.); bala@tacugama.com (B.A.); 5 North Carolina Zoo, Asheboro, NC 27205, USA; 6 Faculty of Medicine, Health and Life Sciences, Swansea University, Swansea SA2 8PP, UK; 7 Cardiff School of Sport and Health Sciences, Cardiff Metropolitan University, Cardiff CF5 2YB, UK

**Keywords:** biomarkers, diet, great apes, triglycerides, cholesterol, HDL, LDL, VLDL, cardiovascular disease

## Abstract

Cholesterol, triglycerides, high-density lipoproteins (HDLs), low-density lipoproteins (LDLs), and very-low-density lipoproteins (VLDLs) were evaluated in chimpanzees at Tacugama Chimpanzee Sanctuary, Sierra Leone. Blood from 75 visually healthy chimpanzees was collected, centrifuged within one hour of collection, and analyzed at Choithram Hospital within 24 h. Statistical analyses assessed differences and interactions based on age, body condition score (BCS), housing group, and sex. HDLs varied widely by housing group; HDLs and LDLs were higher in males than in females. Cholesterol and LDLs were higher in prepubertal individuals while VLDLs and triglycerides were higher in postpubertal individuals. Lipid biomarker differences by age and age ∗ sex statistical interactions were not observed. These data represent a novel compilation of serum lipid biomarkers from a large population of sanctuary-housed Western chimpanzees (*Pan troglodytes verus*) within a range country, a population not previously studied with regard to serum lipid biomarkers. This study has documented significant differences compared to known values from managed chimpanzees and human reference ranges. The relationship of serum lipid biomarkers with health and disease in great apes remains understudied, but the present data set provides a basis for future studies to ascertain whether these differences are healthy biomarker variations or represent an elevated risk factor for disease.

## Simple Summary

Data on the normal concentration of serum lipids in chimpanzees and how these vary among populations, such as human care versus free-ranging, are sparse. This lack of information makes it difficult to understand the relationship between serum lipid dynamics and health problems such as cardiovascular disease in chimpanzees, a prominent cause of death in great apes. Additional data on baseline serum lipids will further our understanding of this relationship. Five lipid biomarkers were evaluated in chimpanzees at Tacugama Chimpanzee Sanctuary (TCS) and compared to managed chimpanzee values (ZIMS). Lipid values from TCS were also compared by age, body condition score, housing group, and sex for the five lipid biomarkers. Blood samples from 75 chimpanzees were collected, centrifuged, and were analyzed within 24 h. Total cholesterol was higher in males than in females and low-density lipoproteins tended to be higher in males. Average very-low-density lipoprotein and triglyceride concentrations exceeded ZIMS values. High-density lipoproteins varied by housing group, possibly due to environment, physical activity, and age. Differences from data published on managed chimpanzees and human reference intervals suggest that chimpanzees at TCS are unique with respect to their lipid values. Future studies are needed to assess whether these differences are healthy variations in the lipid metabolites or represent a higher disease risk.

## 1. Introduction

Chimpanzees are an endangered species of great ape with a declining population, along with a decreasing number of reproductive adults due to anthropogenic factors such as mining, deforestation, habitat fragmentation, and climate change [1]. Even though chimpanzees natively inhabit savannah and forest biomes in 22 countries in sub-Saharan and equatorial Africa [2], many in situ conservation efforts rely on the establishment of sanctuaries or preserves to maintain the chimpanzees’ natural habitat and thus the population. As the global managed population’s mean lifespan exceeds the wild lifespan by 15–20 years [2], chimpanzees under human care may present different nutrient blood parameters compared to their free-ranging counterparts, likely due to more advanced age, but also shifts in diet and/or environment. In contrast to the research supporting a correlation of increased serum lipid biomarkers such as triglycerides and low-density lipoprotein (LDL) with an increased risk of atherosclerosis in humans [3], studies do not support this association in chimpanzees [4,5]. This difference is possibly due to a difference in immune system reactivity between the species [6]. However, it has been shown that managed chimpanzees tend to have higher concentrations of serum lipid biomarkers when compared to their semi free-ranging counterparts [5]. Additionally, sanctuary-housed and semi free-ranging chimpanzee populations have been reported to have lower levels of the serum lipid biomarkers typically associated with cardiovascular disease (CVD) compared to humans [5]. Laboratory and sanctuary-housed chimpanzees’ total cholesterol and triglycerides demonstrate mixed findings regarding the influence of age, sex, or both on CVD [5,7,8]. Given that CVD is globally the main cause of human death [9] and that significant CVD has been documented in great apes [10,11], evaluation of potential risk factors such as lipid biomarkers in chimpanzees may prove beneficial to assessing CVD risk in chimpanzee populations. While unpublished in free-ranging chimpanzees, it has been shown that total cholesterol and LDL in free-ranging gorillas (*Gorilla gorilla gorilla* and *Gorilla beringei*) and Bornean orangutans (*Pongo pygmaeus*) are lower than in their managed counterparts [12]. When there is a lack of serum biochemical normal ranges in great apes, human ranges are often used as reference ranges; however, these values may not all be accurate for great apes. Therefore, characterizing values in chimpanzees and comparing them to humans can provide important comparative data between the two species.

The goals of this study were to (1) characterize chimpanzee serum concentrations of triglycerides, cholesterol, high-density lipoprotein (HDL), low-density lipoprotein (LDL), and very-low-density lipoprotein (VLDL) by age (prepubertal or postpubertal) and sex; (2) compare lipid biomarker concentrations from range country semi free-ranging sanctuary chimpanzees to those from in zoo-housed chimpanzees using Zoological Information Management System (ZIMS) values [13]; and (3) compare these sanctuary population values to published human ranges.

## 2. Materials and Methods

Blood samples, body condition scores (BCSs), body weight, and dietary information were collected during chimpanzee annual wellness exams at Tacugama Chimpanzee Sanctuary (Freetown, Sierra Leone) in late fall of 2024 under full anesthesia. BCSs were on a 1 (very thin) to 5 (obese) scale [14]. Body weight was recorded on the day of the exam via a hanging analog scale with a heavy-duty tarpaulin basket to hold each individual, as soon as chimpanzees were anesthetized. The study population included 48 females (38 of which were on progestin implant contraception) and 29 males, one of which was castrated. Chimpanzees ranged in age from 3 to 41 years. Additionally, the study group was divided into age classes and sex as follows: 15 prepubertal females, 13 prepubertal males, 29 postpubertal females, and 18 postpubertal males. In this study, age categories used were as previously defined [15]: infants (<6yr,) juveniles (6 yr < 13 yr), adults (13 yr < 40 yr), and geriatrics (40 yr). For statistical analysis, infants and juveniles were combined (prepubertal), and adults and geriatrics were combined (postpubertal) (Table 1 and Table 2). Chimpanzees were housed on a single property in eight housing units (Table 1) with group size ranging from 1 to 18 individuals and varied by age to facilitate post-rescue management and integration into normal social units. For example, infants that required round-the-clock care and feeding were housed together. Three of the groups (groups 1, 2, and 8) included temporarily individually housed animals due to high security risks, medical concerns, or age-related restrictions. Daily dietary data were collected via keeper feed logs as well as TCS purchase records during the fall of 2024. Researchers participated in daily feedings to examine diet ingredients and distribution methods as time permitted. Researchers examined diet sheets and accompanied keepers on numerous feedings to observe the precise administration of feed items rather than solely relying on diet sheets and recipes.

Anesthesia was administered at the discretion of the attending veterinarian using various combinations of medetomidine HCl (10 mg/mL, Chiron Pharmacy Inc., Guelph, ON, Canada), tiletamine-zolazepam, (Zoletil^®^ 100, Virbac, Milperra, Australia), ketamine HCl (USO 50 mg/mL, Citron Drugs and Pharmaceuticals Pvt. Ltd., Tarapur, India), and/or midazolam 1 mg/mL (Hameln Pharma Ltd., Gloucester, UK). Depending on the individual chimpanzee, intramuscular injections were delivered via hand injection or via dart. While monitoring vitals under anesthesia, blood samples were drawn from the femoral vein into Vacutainer^®^ serum separator tubes and centrifuged within an hour of collection; serum was refrigerated and then transported on ice to a local human hospital (Choithram Memorial Hospital—Freetown, Sierra Leone) where they were analyzed within 24 h of collection. At the time of anesthesia and sample collection, all animals had fasted for 15–22 h (with access to water). Laboratory results were electronically returned to the veterinary medical care team and entered into the commercial statistical software (SAS, Cary, NC, USA) for statistical analysis. https://www.sas.com/zh_cn/home.html

Choithram Memorial hospital used a fully automated clinical chemistry analyzer (Erba Manheim EM 200, Erba, Dubai Science Park, Dubai, UAE) to measure the lipid biomarkers. Cholesterol, HDL, LDL, and triglycerides were directly measured, while VLDL was calculated from triglycerides using the Friedewald equation [16].

### Statistical Analysis

A Proc GLM ANOVA was used to identify any association between the chimpanzee housing group (which varied by individual demographics of age, location, and environment) and lipid value (cholesterol, HDL, LDL, VLDL, triglycerides) least square (LS) means (a = 0.05). Additionally, a separate Proc GLM ANOVA was used to determine if sex or age category (prepubertal or postpubertal) or sex * age category interactions altered lipid biomarker LS means. The results were analyzed by comparing all eight groups and also the five groups that did not have individually housed animals. Finally, a separate Proc GLM ANOVA was run to determine if BCS affected cholesterol, HDL, LDL, VLDL, or triglycerides.

Values for the TCS population were compared to those for managed populations by using Zoological Information Management System (ZIMS) reference intervals (accessed on 19 November 2024). Evaluation was conducted by marking any TCS value outside of the ZIMS range as irregular and calculating the percentage of individuals with values outside the ZIMS range for each of the five lipid biomarkers. Comparisons were made between TCS chimpanzees and ZIMS reference intervals for all lipid biomarkers except VLDL, which was compared against the values of serum or plasma VLDL, as grouped in the system. Chimpanzee values from the present study were also compared to published human normal values and Choithram Hospital laboratory human reference values [17,18,19]. In instances where human reference values followed a tiered system (such as giving values for healthy, at-risk, or dangerous) or were inconsistent, the higher value was utilized as the threshold.

## 3. Results

The TCS chimpanzee population overall was deemed healthy by the attending veterinarians. The mean ± SD body condition score (BCS) was 3.1 ± 0.43. The median was 3.0, with a range of 2.0–4.5. None of the lipid biomarkers statistically differed by BCS.

The mean ± SD body weight for the entire TCS population was 38.2 ± 11.45 kg. The mean body weight for males was 37.8 ± 14.51 and for females 38.5 ± 8.85. Mean prepubertal body weight was 28.4 ± 12.05 and postpubertal body weight was 44.1 ± 5.63. Due to the confounding nature of the BCS and/or age, lipid biomarkers were not statistically analyzed by weight.

### 3.1. Comparison by Housing Group

Only HDL was significantly different by housing group (*p* = 0.0002) (Table 1). This was significant when all eight groups were compared as well as when the three groups with individually housed animals were excluded. While HDL values differed by housing group, a clear pattern was not discernible by sex or age.

### 3.2. Comparison by Age and Sex

When sex effects were examined separately, LDL was lower in females (*p* = 0.03) and HDL tended to be lower in females (*p* = 0.08) (Table 2). There were no age ∗ sex interactions when lipid biomarker concentrations were analyzed.

When age effects were examined separately, LDL was higher (*p* = 0.05) and cholesterol tended to be higher (*p* = 0.10) in prepubertal compared to postpubertal chimpanzees (Table 2). VLDL and triglycerides were lower in prepubertal than postpubertal chimpanzees (*p* = 0.05 for both).

### 3.3. ZIMS Comparison

Individual values for HDL and LDL were within the range of the ZIMS reference interval (RI) [13], with zero outliers and 100% overlap. Cholesterol had only two values outside of the ZIMS RI. In contrast, both triglycerides and VLDL were significantly different from the ZIMS RI, with 51 triglyceride values above the reference range and 47 VLDL values above the reference range. Of the 75 individuals assessed, 23 were within ZIMS RI for all five lipid biomarkers. LDL and cholesterol were the only lipid biomarkers from the TCS population to be within one standard error of the corresponding means reported by ZIMS (Table 3).

### 3.4. Human Comparison

Similar to the comparison with ZIMS, cholesterol, HDL, and LDL had the greatest overlap with the human range: 70.2, 78.4, and 60.8% overlap, respectively. The lowest overlap was for VLDL, where only 16.7% of TCS chimpanzees had values within the normal human reference range. The overlap of TCS values with the human range for triglycerides was only 21.6%. None of the five biomarkers assessed were more than 90% within the human reference value range, and of the 75 individuals assessed, five (6.7%) were completely within the human reference range for each biomarker assessed (Table 3).

### 3.5. Diet Information

Dietary information was collected from TCS records and keeper records, as there was some variation between the TCS records and what was actually fed. This discrepancy occurred primarily due to the availability of food items (Table 4). Since the chimpanzees were housed in eight groups that were fed by several different staff members, diet items and quantities varied notably across individuals within this study.

## 4. Discussion

With a sample size of 75 sanctuary-housed Western chimpanzees within a range country, this study has one of the largest datasets of chimpanzee lipid profiles to date. TCS lipid biomarker values differed markedly from values for zoo-housed chimpanzee populations, based on ZIMS global data. Interestingly, TCS lipid values for all five lipid biomarkers were higher than the values reported by Cole et al., even though both populations are sanctuary-based chimpanzees, albeit likely different subspecies [5]. The present study is in agreement with previous studies in humans which showed that diet, sex, age, and lifestyle can affect serum lipids [20,21,22]. While the current results did not show a difference in the lipid biomarkers associated with BCS, this was likely due to the fact that the TCS chimpanzee population was physically fit with limited number of overweight and underweight animals (mean BCS = 3.1 on of a 5-point scale). It should be noted that the current TCS adult population body weights (38.5 to 37.8 kg, males to females, respectively throughout) are lower than those of sanctuary (43.5–52.6 kg), zoo (54.7–61.8 kg), and research chimpanzees (58.7–63.8 kg) despite the noted elevated lipid metabolite values [23].

There were considerable differences when comparing the current total cholesterol and triglyceride data with another range country sanctuary project previously conducted at Tchimpounga Chimpanzee Rehabilitation Centre (TCRC), the Republic of the Congo. Total cholesterol was notably lower at TCRC (3.95 mmol/L) (n = 44) [21] compared to our study population (4.8 mmol/L). The TCRC overall population triglyceride values (0.46 mmol/L and 1.27 mmol/L measured at two different laboratories) [21] were much lower than the TCS overall population value (3.8 mmol/L). Both sanctuary populations were approximately 40% male, although the TCS population age range was wider (3 to 41 years old) compared to TCRC (6 to 28 years of age). The chimpanzee subspecies differed between the two populations with *Pan troglodytes verus* at TCS and *Pan troglodytes troglodytes* at TCRC, and these subspecies have previously shown differences in lipoprotein (a), with *P. t. verus* having higher values [8]. However, while subspecies variation could contribute to these differences, the available data (drawn largely from *P. t. verus* and *P. t. troglodytes* populations in two range country sanctuaries) are too limited to determine whether this is the case [8].

HDL is higher in more physically active individuals, which corresponds to the housing group HDL comparisons [20]. Differences in physical activity may explain differences in HDL cholesterol between groups, as some groups had the ability to exercise more than others. Additionally, some of the groups contained a more skewed population by age, with group 8 having two very young individuals and high HDL, while group 1 had two geriatric individuals and much lower HDL. Age also co-varies with physical activity [22,24], and thus the variation in HDL among groups could be associated with differences age itself or with the differences in exercise as a function of age and housing type. Additionally, the keeper staff assigned to each group differed, and diet may have also varied between groups, although technically the recommended daily diet was the same. It is noteworthy that the grain balls fed daily to the chimpanzees are 7% palm oil. While palm oil has been associated with elevated total cholesterol, HDL, and LDL, this association is tentative and it is unlikely that the results noted for the TCS population were due to palm oil consumption alone, especially considering the small amount it contributed to the total daily diet quantity [25].

It is difficult to ascertain why triglyceride and VLDL concentrations differ between the TCS and ZIMS populations, but cholesterol, HDL, and LDL do not. Tests for the normality of the distribution of the biomarkers for all individuals documented that LDL values were skewed right, with more individuals having lower LDL values and very few individuals having high LDL values. The ZIMS data encompass VLDL measurements from 61 individuals and triglyceride measurements from 370 individuals; therefore, sample size differences may have affected the results. As expected, serum concentrations of triglycerides and VLDL in TCS chimpanzees were more similar to those from ZIMS chimpanzees than to human ranges. Future studies coupled with dietary analysis are needed to assess the biochemical sources of these differences. Additionally, since VLDL was calculated value from triglycerides it is not a surprise that their results follow a similar pattern. Additionally, the Friedewald VLDL equation is known to be less reliable (potentially overestimated) when triglyceride values are very high (over 4.5 mmol/L) [26]. Since one-third of our chimpanzee population had triglyceride values over 4.5, it is possible that our reported VLDL values are slightly overestimated.

Although the relationship between circulating lipid markers and cardiovascular health in chimpanzees is likely distinct from that in humans [4,5], given that chimpanzees rarely develop atherosclerosis, elevated lipid levels may still hold clinical relevance. In chimpanzees, the leading cause of cardiac mortality is diffuse interstitial myocardial fibrosis (IMF), a condition with an as-yet uncertain etiology [27]. While the mechanisms underlying IMF remain unclear, elevated lipid concentrations could plausibly contribute through pathways involving oxidative stress and chronic inflammation [28].

Therefore, it is crucial to establish lipid biomarker ranges across chimpanzee populations and environments to determine whether elevated concentrations are clinically relevant and whether they may influence the development of IMF. Much remains to be clarified about the pathogenesis of CVD in chimpanzees. For example, atherosclerosis has been reported both in cases of idiopathic cardiomyopathy and in chimpanzees without cardiac disease, whereas individuals with other forms of heart disease have shown no evidence of atherosclerosis [29]. Thus, although it remains uncertain whether the lipid biomarker values observed truly constitute hyperlipidemia in chimpanzees, based on human research, persistently elevated serum lipids could contribute to conditions such as hypertension, idiopathic cardiomyopathy, atherosclerosis, myocardial infarction, and stroke [30,31]. While direct evidence in chimpanzees is lacking, these pathways remain biologically possible and represent an important area for future research.

Dietary cholesterol comes from animal products in the diet [32]. Although a complete dietary profile of the studied chimpanzees was not available (Table 4), the only animal products provided in the diet were eggs and milk, of which eggs are naturally high in cholesterol [32]. The level of egg intake needed to provide elevated cholesterol among most of the 75 individuals was not observed. Another source of animal products for free-ranging chimpanzees is hunting, and although some of the chimpanzees have a sufficiently large enclosure to exercise, they are unlikely to exhibit species-typical hunting behaviors, which would provide substantial sources of animal products. A future complete feedstuff breakdown by exact feed items and grams fed with comprehensive dietary evaluation would help delineate possible causes for elevated lipids and determine the impacts of diet, environment, and natural foraging behaviors in this group.

These data have been analyzed by housing type, but a more thorough analysis of true differences between enclosures is needed. While some enclosures included significant and expansive free-ranging habitat, others were limited to little-to-no naturally forested habitat. Activity level may also play a role in the lipid biomarker values reported, which would be affected by the housing type and age of the members in the group, as noted above. Additionally, while an introductory diet analysis is provided, it is vital to elucidate the true differences in feeding patterns between groups, and perhaps between keepers, which could explain some of the differences by group, as reported in the present study. All these factors have the potential to be confounding variables, and further research and data analysis will help elucidate the potential sources of cholesterol as well as better establish what is normal before providing stronger recommendations for chimpanzee dietary management with respect to serum lipid biomarker profiles. Furthermore, even though all lipid samples were analyzed with the same laboratory equipment at a human hospital, factors such as transit time, ambient temperature, and equipment differences may have played a minor role in influencing results, which could be compared in the future [26,33,34,35]. It should be noted that ZIMS reference intervals are calculated based on samples from animals reported by their institutions as healthy by each chimpanzee’s attending veterinarian. Therefore, ZIMS data may include subclinical disease, obesity (as obesity is not considered a disease by ZIMS), and/or individuals sampled multiple times, but ZIMS remains the most comprehensive dataset available [13]. Comparing human data from the same hospital where the chimpanzee lipid samples were analyzed may prove informative. While chimpanzee serum lipid values have been studied in laboratory-housed, zoo-housed, and sanctuary-housed populations, the current study provides novel data from a large and previously unpublished population, yielding results that significantly differ from published results.

## 5. Conclusions

In summary, this study reports the first published serum lipid biomarker values for sanctuary-housed *Pan troglodytes verus*. In this population, males had higher cholesterol and LDL values than females. Prepubertal chimpanzees had higher cholesterol and LDL values than postpubertal chimpanzees but lower triglycerides and VLDL values. HDL varied by housing group and was potentially linked to age, environment, and small variations in diet between groups. Notably, this population of sanctuary chimpanzees had higher triglycerides and VLDL values than reported in zoo-housed chimpanzee data (ZIMS) and human reference ranges.

It may be beneficial to explore the genetic and subspecies factors on serum lipid biomarkers in future studies, as well as to compare the biochemical processes associated with lipid metabolism and specifically the five metabolites discussed, in chimpanzees versus humans in order to better understand the comparative aspects of the etiology of cardiovascular disease in the two species.

## Figures and Tables

**Table 1:** Tacugama Chimpanzee Sanctuary, Sierra Leone, housing groups, including the number of individuals in each group (n), age category (prepubertal or postpubertal), sex, and high-density lipoprotein (HDL) LS means. HDL mean differences by group are also presented ^1^.

Group Number ^2^	Individual Sex and Age Group	HDL (mmol/L) ^1^
1 (n = 2)	2 postpubertal males	1.8 ± 0.34 ^a,b,c,d^
2 (n = 6)	3 postpubertal males and 3 postpubertal females	2.1 ± 0.20 ^a,d^
3 (n = 13)	3 postpubertal males and 10 postpubertal females	2.2 ± 0.13 ^a,c^
4 (n = 13)	6 males (3 postpubertal, 3 prepubertal) and 7 females (5 postpubertal, 2 prepubertal)	2.4 ± 0.13 ^a,c^
5 (n = 10)	4 males (1 postpubertal, 3 prepubertal) and 6 females (2 postpubertal, 4 prepubertal)	1.6 ± 0.15 ^b^
6 (n = 11) ^3^	6 males (2 postpubertal, 4 prepubertal) and 5 prepubertal females	1.6 ± 0.15 ^b,d^
7 (n = 18) ^3^	6 males (4 postpubertal, 2 prepubertal) and 12 females (8 postpubertal, 4 prepubertal)	1.9 ± 0.11 ^b,c^
8 (n = 2)	2 prepubertal females	2.2 ± 0.34 ^a,b,c,d^

^1^ Groups with common superscripts are not significantly different (*p* < 0.05). ^2^ Individuals (n) in each group. ^3^ All postpubertal animals in groups 6 and 7 were close to puberty (13 years old).

**Table 2:** Lipid biomarker LS means for Tacugama Chimpanzee Sanctuary chimpanzees ± standard error of the means by sex (female or male) and age (prepubertal or postpubertal) ^1,2^.

	Cholesterol (mmol/L)	HDL (mmol/L)	LDL (mmol/L)	VLDL (mmol/L)	Triglycerides (mmol/L)
Female (n = 48) ^3^	4.6 ± 0.16	1.9 ± 0.09 ^x^	2.4 ± 0.10 ^a^	1.6 ± 0.12	3.5 ± 0.26
Male (n = 29)	5.0 ± 0.19	2.1 ± 0.10 ^y^	2.8 ± 0.12 ^b^	1.9 ± 0.14	4.1 ± 0.31
Prepubertal (n = 28)	5.0 ± 0.20 ^x^	1.9 ± 0.10	2.8 ± 0.13 ^a^	1.5 ± 0.15 ^a^	3.3 ± 0.33 ^a^
Postpubertal (n = 49)	4.6 ± 0.15 ^y^	2.0 ± 0.08	2.4 ± 0.10 ^b^	1.9 ± 0.12 ^b^	4.1 ± 0.25 ^b^

^1^ Category LS means with different superscripts a, b differ statistically (*p* < 0.05). ^2^ Category LS means with different superscripts x, y trend to differ (*p* = 0.05–0.10). ^3^ Number (n) of individuals in each grouping.

**Table 3:** Summary of serum lipid biomarkers for chimpanzees (*Pan troglodytes*) from Tacugama Chimpanzee Sanctuary (TCS) (n = 75) in Freetown, Sierra Leone, from zoo-housed populations [Zoo Information Management System global reference intervals (ZIMS)] and published human reference values. All values are reported in mmol/L.

Lipid Biomarker	TCS Mean ± Standard Error	ZIMS Mean ^1^	ZIMS Reference Interval ^1^	Human Reference Value
Triglycerides	3.8 ± 0.20	1.13	0.37–2.59 (n = 370)	<1.69 ^2^
Cholesterol	4.8 ± 0.13	4.84	2.64–7.69 (n = 581)	<5.17 ^3^
High-Density Lipoprotein	2.0 ± 0.06	1.31	0.39–2.85 (n = 95)	>1.55 ^2^
Low-Density Lipoprotein	2.6 ± 0.08	2.59	0.89–5.08 (n = 60)	<2.59 ^2^
Very-Low-Density Lipoprotein	1.8 ± 0.09	0.66	0.22–1.56 (n = 61)	<0.78 ^4^

^1^ All ZIMS data were procured from a 19 November 2024 system report [13]. Data reported are from serum samples, with the exception of VLDL, which is reported from the “serum or plasma” samples category, as serum samples did not have a reference interval. ^2^ See reference [17]. ^3^ See reference [18]. ^4^ See reference [19].

**Table 4:** General dietary frequency and quantity of food groups fed to the chimpanzee groups at Tacugama Chimpanzee Sanctuary (TCS) in Freetown, Sierra Leone.

Food Group	Chimpanzee Groups Fed	Frequency	Quantity Per Chimpanzee
Grain balls ^1^	All	1 time daily	2–3 balls (~75 g each)
Browse ^2^	Large Enclosures	unknown	unknown
Eggs (hard boiled)	All	3 times weekly	1 egg
Enrichment ^3^	All	1 time daily	~1 cup
Ginger tea ^4^	All	1 time daily	~1 cup
Milk ^5^	Age and Medical	1–2 times daily	0.25–0.75 cup
Seasonal produce ^6^	All	2 times daily	Varied
Sweet potato	All	1 time daily	Varied (0.5−75 kg)

^1^ Balls are approximately 54% garri (cassava), 10.3% beans, 7% white rice, 10.3% ground nuts, 10.3% oats, and 7% palm oil. Each chimpanzee receives two balls, with alpha animals occasionally getting three. ^2^ Some chimps are housed in enclosures with free access to new and old forest growth during the day. ^3^ Includes two to three of the following: jam, biscuits, crackers, ground nuts, popcorn, spaghetti, apples, garlic, cornflakes, dates, sesame seeds, cabbage, carrots, and spring onions. ^4^ Fresh ginger tea, honey, and lime. Cup size and ingredient composition varied by keeper. ^5^ Instant Fat-Filled Powder. At each offering, each keeper fills a bucket with 10 scoops (~85 g) of milk powder, but the amount of water varies, resulting in varying concentrations of milk. Cup size is also variable. Only chimpanzees categorized as young, old, or medically vulnerable were provided milk. ^6^ Includes banana, cabbage, chuk chuk plum, coconut, corn, cucumber, eggplant, mango, grapefruit, guava, locust bean, molumbo plum, okra, orange, palm nuts, papaya, pineapple (with or without the crown), potato leaf, pumpkin, rough-haired plum, sugar cane, and watermelon. TCS attempts to feed only one high-sugar fruit item per day, and this is availability-dependent.

## Data Availability

The data presented in this study are only available on request from the corresponding author due to official confidentiality restrictions.

## Abbreviations

The following abbreviations are used in this manuscript:

CVD	Cardiovascular disease
HDL	High-density lipoprotein
LDL	Low-density lipoprotein
NIH	National Institutes of Health
SAS	Statistical Analysis System
SEM	Standard error of the mean
TCS	Tacugama Chimpanzee Sanctuary
TCRC	Tchimpounga Chimpanzee Rehabilitation Centre
VLDL	Very-low-density lipoprotein
ZIMS	Zoological Information Management Systems
